# Chronic Administration of 7,8-DHF Lessens the Depression-like Behavior of Juvenile Mild Traumatic Brain Injury Treated Rats at Their Adult Age

**DOI:** 10.3390/pharmaceutics13122169

**Published:** 2021-12-16

**Authors:** Shih-Te Yang, Hsiu-Yi Hung, Long-Sun Ro, Ming-Feng Liao, Tamara G. Amstislavskaya, Maria A. Tikhonova, Yi-Ling Yang, Kwok-Tung Lu

**Affiliations:** 1 Department of Life Science, National Taiwan Normal University, Taipei 11677, Taiwan; hello9300102a@gmail.com (S.-T.Y.); h82113joe@gmail.com (H.-Y.H.); mingfengliao@hotmail.com (M.-F.L.); 2 Department of Neurology, Chang Gung Memorial Hospital-Linkou Medical Center and Chang Gung University College of Medicine, Taipei 33305, Taiwan; cgrols@adm.cgmh.org.tw; 3 Institute of Medicine and Psychology, Novosibirsk State University, 630090 Novosibirsk, Russia; amstislavskayatg@physiol.ru; 4 Laboratory of Experimental Models of Neurodegenerative Processes, Scientific Research Institute of Neurosciences and Medicine, 630117 Novosibirsk, Russia; tikhonovama@physiol.ru; 5 Department of Biochemical Science and Technology, National Chiayi University, Chiayi 600355, Taiwan

**Keywords:** juvenile, mild traumatic brain injury, depression-like behavior, ventral hippocampus, dorsal hippocampus

## Abstract

Traumatic brain injury (TBI) is a leading cause of mortality and morbidity among the global youth and commonly results in long-lasting sequelae, including paralysis, epilepsy, and a host of mental disorders such as major depressive disorder. Previous studies were mainly focused on severe TBI as it occurs in adults. This study explored the long-term adverse effect of mild TBI in juvenile animals (mTBI-J). Male Sprague Dawley rats received mTBI-J or sham treatment at six weeks old, then underwent behavioral, biochemical, and histological experiments three weeks later (at nine weeks old). TTC staining, H&E staining, and brain edema measurement were applied to evaluate the mTBI-J induced cerebral damage. The forced swimming test (FST) and sucrose preference test (SPT) were applied for measuring depression-like behavior. The locomotor activity test (LAT) was performed to examine mTBI-J treatment effects on motor function. After the behavioral experiments, the dorsal hippocampus (dHip) and ventral hippocampus (vHip) were dissected out for western blotting to examine the expression of brain-derived neurotrophic factor (BDNF) and tropomyosin receptor kinase B (TrkB). Finally, a TrkB agonist 7,8-DHF was injected intraperitoneally to evaluate its therapeutic effect on the mTBI-J induced behavioral abnormalities at the early adult age. Results showed that a mild brain edema occurred, but no significant neural damage was found in the mTBI-J treated animals. In addition, a significant increase of depression-like behaviors was observed in the mTBI-J treated animals; the FST revealed an increase in immobility, and a decrease in sucrose consumption was found in the mTBI-J treated animals. There were no differences observed in the total distance traveled of the LAT and the fall latency of the rotarod test. The hippocampal BDNF expression, but not the TrkB, were significantly reduced in mTBI-J, and the mTBI-J treatment-induced depression-like behavior was lessened after four weeks of 7,8-DHF administration. Collectively, these results indicate that even a mild juvenile TBI treatment that did not produce motor deficits or significant histological damage could have a long-term adverse effect that could be sustained to adulthood, which raises the depression-like behavior in the adult age. In addition, chronic administration of 7,8-DHF lessens the mTBI-J treatment-induced depression-like behaviors in adult rats. We suggest the potential usage of 7,8-DHF as a therapeutic agent for preventing the long-term adverse effect of mTBI-J.

## 1. Introduction

Traumatic brain injury (TBI) is a leading cause of mortality and morbidity among the global youth [1,2,3,4]. Over the past two decades, the mortality of TBI in both the United States and Taiwan has dropped, which could be attributed to law enforcement efforts, advances in sports safety equipment, more accessible access to first aid, and improvements to diagnostic techniques [5,6,7]. With the increased survival rate also came an influx of TBI-related psychiatric cases [5,6,7,8,9,10].

The brain damage induced by TBI is a complex series of events best described as four overlapping phases: primary injury, a progression of injury, secondary damage, and regeneration [11,12,13,14]. Primary injury to the brain may result from: (1) brain contusion via direct skull impact; (2) brain abrasion against the rough interior surface of the skull; (3) shearing and stretching of brain tissue via violent motion; or (4) vascular response to impact, including subdural hematoma characterized by the rupture of blood vessels located between the brain and dura mater.

The pathophysiology of TBI is amplified by secondary injuries such as hypoxia, ischemia, increased intracranial pressure, seizure, and cerebral edema. Recent studies have heavily focused on preventing or at least ameliorating secondary injury as a means to delay brain damage. Specific targets such as the glutaminergic neurotransmission systems, free radical production [15], lipid peroxidation [16], growth factors [17], inflammatory cytokines [14,18,19], and ion transport [20,21,22] have been identified.

The cerebral cortices (e.g., prefrontal and temporal cortex) are at high risk of being damaged under TBI. Brain damage from TBI may result from rotational (angular) force, linear (translational) force, or the blunt force of deceleration upon impact, leading to single or multiple contrecoup contusions [23,24]. However, the subcortical structures such as the limbic system (hippocampus, amygdala, nucleus accumbens) are also susceptible to such impact [23,24], which may explain that TBI patients tend to suffer from emotional disorders and memory-related diseases [25,26,27]. TBI patients often suffer from major depressive disorder (MDD) [27,28,29,30,31], posttraumatic stress disorder (PTSD) [24,32,33,34], and both anterograde and retrograde amnesia [35,36].

Brain edema is the most common and severe secondary injury associated with TBI, which could elevate intracranial pressure and result in death. Generalized anxiety disorder (GAD), PTSD, and MDD are the most prevalent psychiatric sequelae of TBI. Previous TBI studies have focused mainly on adults [37,38,39], but adolescent males comprise the largest TBI cohort with 21.3% of total cases [40]. Therefore, in the urgent interest of public health, researchers are beginning to focus on elucidating the pathological mechanisms and devising new therapies for the neurological and psychological consequences of adolescent TBI. 

Major depressive disorder (MDD) is one of the most frequently reported conditions associated with TBI, with a prevalence of 25% to 40% in mild-to-severe TBI cases compared to a 17% prevalence in the general population [41,42]. In one prospective, multicenter study involving 666 cases of moderate-to-severe TBI, high rates of several depression-like symptoms including fatigue (29%), distractibility (28%), anger/irritability (28%), and rumination (25%) were found [43]. Almost 27% of TBI patients diagnosed with depression in this study experienced feelings of hopelessness, worthlessness, and anhedonia. Past research has suggested that MDD may result from the abnormal expression/phosphorylation of amygdaloid mitogen-activated protein kinase (MAPK) or tropomyosin receptor kinase B (TrkB)—both respectively induced by acute and chronic stress [17,44,45,46]. Elevated amygdaloid long-term potentiation (LTP) has also been observed in MDD animal models [47,48]. Our pilot experiment showed that both increases of depression-like behavior and elevated amygdaloid LTP were found in the juvenile mild TBI-treated rats (mTBI-J), which suggests our model could effectively mimic the pathological condition in TBI patients. 

The tropomyosin receptor kinase B (TrkB) is the brain-derived neurotrophic factor (BDNF) receptor. This is abundantly expressed in the central nervous system, particularly the limbic system, including the hippocampus and amygdala. Previous results demonstrated that the hippocampal and amygdaloid BDNF expression level is inversely related to the severity of MDD. The efficacy of antidepressants is positively correlated with their modulation effect at the BDNF expression [49], and both selective serotonin reuptake inhibitors (SSRIs) and tricyclic antidepressants (TCAs) normalize BDNF expression in MDD patients [50,51]. Additionally, electroconvulsive treatment (ECT) may reduce depressive symptoms by increasing the expression of BDNF in the hippocampus [52]. Conclusively, these results suggest BDNF is an essential factor for the pathological mechanism of MDD. 

It was recently demonstrated that 7,8-dihydroxyflavone (7,8-DHF), a novel stroke medication permeable to the membrane and able to cross the blood–brain barrier (BBB), acts as a BDNF mimetic to reduce depression-like behavior in mice and rats [53,54]. Other research has found that 7,8-DHF functions as an antioxidant to protect cells from apoptosis [55] and even prevent synaptic loss associated with Alzheimer’s disease [56]. The present study also investigates the possible therapeutic benefits of 7,8-DHF by a TBI-induced depression model. 

It is well-known that the abundance of excitatory neurotransmission within the hippocampus makes it vulnerable to stroke and traumatic brain injury [57]. It has been reported that the ventral hippocampus plays an important role in depression-like syndromes [58]. We suggested that juvenile TBI treatment may have long-term regulation effects on the hippocampal BDNF and TrkB expression, leading to increased depression-like behavior in adulthood. The present study is aimed to examine this hypothesis.

## 2. Materials and Methods

### 2.1. Animals

Juvenile male Wistar rats purchased from BioLASCO Co., Ltd. (Taipei, Taiwan). were kept at a temperature of 24 ± 1 °C, in a well-ventilated vivarium, with a 12/12 daylight cycle and access to water and food *ad libitum* at all times. All procedures were adapted from the National Institutes of Health Guide for Care and Use of Laboratory Animals and the guidelines set forth by the IACUC at the National Taiwan Normal University and carried out following the ARRIVE guidelines (IACUC Approval Number: 109046). All efforts were made to minimize the animals’ suffering and use fewer animals. 

### 2.2. The Juvenile Mild Traumatic Brain Injury Model

**Figure 1:**
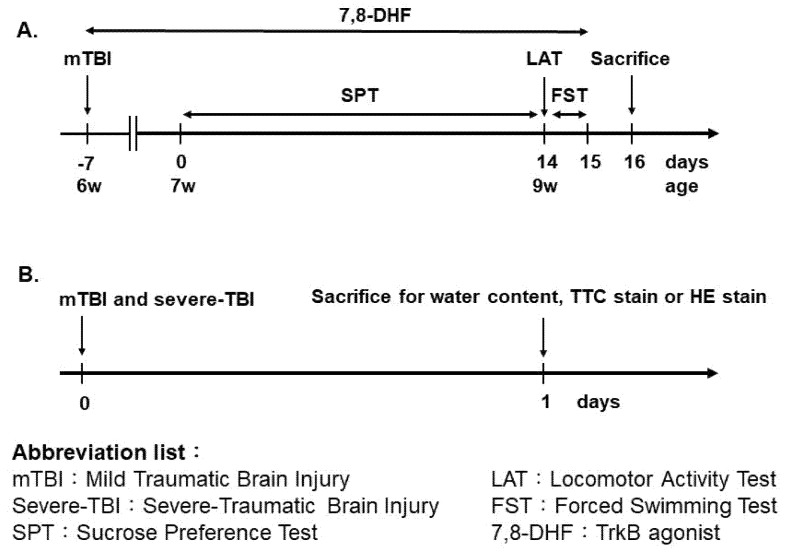
Timeline for the experimental procedure. (**A**) General experimental procedure for evaluating the depression-like behavior in mTBI treated animals. (**B**) The experimental procedure for evaluating the brain damage in mTBI and severe-TBI treated animals.

A modified Marmarou’s weight drop model was applied to induce mild impact acceleration to diffuse brain injury [59]. While under anesthesia by pentobarbital (50 mg/kg, intraperitoneally), a midline incision was made in the scalp, and adjacent skin flaps were opened laterally to expose the skull. A metal helmet was then temporarily placed over the skull to prevent fracture. Next, rats were placed in the prone position under the force delivery device, and a 150-g weight was allowed to fall freely from a height of 0.5 m onto the metal helmet [60]. All animals were allowed to recover from surgery for seven days before behavioral testing. For evaluating the therapeutic effect of TrkB agonist 7,8-DHF, animals received their first 7,8-DHF (5 mg/kg, i.p.) injection immediately after the mTBI-J treatment, once a day for a total of twenty-eight days (Figure 1).

### 2.3. Brain Damage Measurement

a. 2,3,5,-triphenyltetrazolium chloride monohydrate stain (TTC stain)

Twenty-four hours after mTBI-J treatment, rats were anesthetized with 100 mg/kg pentobarbital and then decapitated. Brains were quickly removed, and 2 mm coronal slices were made with a rodent brain matrix. Sections were stained for 20 min with 2% 2, 3, 5,-triphenyltetrazolium chloride monohydrate (TTC) (Sigma, Marlborough, MA, USA) at 37 °C. In a double-blinded manner, the infarction volume was analyzed by ImageJ analysis software. Sections were scanned and the contusion volume in 8–12 mm coronal slices, then set up the threshold to select the complete corpus callosum by ImageJ image processing. White volume on 8–12 mm section was summed and multiplied by section thickness to give the white volume and percentage [20,61,62,63].

b. Brain edema

Animals were anesthetized with 100 mg/kg pentobarbital and then decapitated 24 h after TBI treatment. Brains were quickly removed and weighed. Subsequently, each brain was desiccated at 70 °C for 48 h. Reweighing took place to obtain the dry brain weight. By subtracting from the total brain weight, the wet weight of the brain was obtained. Water content was determined as a percentage of the total brain weight and calculated according to the following formula [64]:

$$\%\;\mathrm{Water}\;\mathrm{content}\;=\frac{100\;\times \;\left(\mathrm{wet}\;\mathrm{weight}-\mathrm{dry}\;\mathrm{weight}\right)}{\mathrm{wet}\;\mathrm{weight}}$$

### 2.4. Locomotor Activity Test (LAT)

For measuring locomotor activity, rats were placed in the center of a testing chamber with a base 42 cm by 42 cm and a height of 36 cm. Rats were then left to move about freely for 10 min. The total moving distance during the test stage was recorded and analyzed using Smart 3.0 software (Panlab, San Diego, CA, USA)

### 2.5. Sucrose Preference Test (SPT)

The first two days of this test involved an acclimation stage during which rats were presented with a choice of either regular water or a 1% sucrose solution. During this period, the rats learned which bottle contains which fluid, and then formed preferences measurable by the amount of fluid consumed. The position of water bottles was switched daily to ensure that the rats chose based on sucrose rather than purely by habit. Testing periods lasted for 12 days. After the data was collected, the sucrose preference ratio was calculated according to the following formula:

$$\mathrm{Preference}\;\mathrm{ratio}\;=\frac{\mathrm{sucrose}\;\mathrm{water}\;\mathrm{intake}}{\mathrm{total}\;\mathrm{water}\;\mathrm{intake}}$$

### 2.6. Forced Swim Test (FST)

We used a modified Porsolt protocol [65] for the forced swimming test, during which a swimming cylinder 18 cm in diameter and 45 cm in height was filled 36 cm deep with water—deep enough that rats were unable to touch the bottom with their feet or tail without falling below the surface. Water temperature was maintained at 25 ± 1 °C. The first day consisted of an acclimation stage during which rats were forced to swim continuously for 15 min. Afterwards, rats were placed into a box containing a 60 W lightbulb for 30 min to dry before returning to their home cages. The test stage was conducted 24 h following the acclimation stage. Rats were forced to swim for 5 min during the test stage while the video was taken and analyzed using Smart 3.0 software (Panlab, San Diego, CA, USA). The percent time of immobility was recorded and analyzed as an index of depression-like behavior. 

### 2.7. Western Blot

Following behavioral experimentation, rats were sacrificed, and their brains were promptly placed onto dry ice. The dorsal and ventral hippocampus were dissected and homogenized in a tissue extraction buffer (Tissue Extraction Reagent Invitrogen™, Waltham, MA, USA). Samples were then centrifuged at 13,200 rpm for 30 min at 4 °C. The total protein concentration was determined using a Bio-Rad Bradford Protein Assay Kit (Bio-Rad, Hercules, CA, USA). A total of 25 μg of protein from each sample was electrophoresed on SDS-PAGE then separated on a PVDF membrane (Merck Millipore, Darmstadt, Hesse, Germany). 

Blotting was blocked in PBS containing 5% skim milk overnight at 4 °C. Primary antibodies were selected for BDNF (1:1000; Abcam, Inc., Cambridge, UK), TrkB (1:1000; Cell Signaling Tech, Inc., Danvers, MA, USA), and GAPDH, (1:5000; Abcam, Inc., Cambridge, UK). The PVDF membranes were kept in a primary antibody TBST solution overnight at 4 °C before adding HRP-conjugated secondary antibodies (1:5000; Abcam, Inc., Cambridge, UK) for 1 h at 4 °C. Binding was detected using enhanced chemiluminescence (Bioman Scientific Co. Ltd., New Taipei City, Taiwan) and was recorded by photographic film (Fujifilm, Tokyo, Japan). Evidence of protein presence was analyzed by ImageJ analysis software (National Institutes of Health, Bethesda, MD, USA).

### 2.8. Statistics

Data are presented as means ± SEM. Using unpaired *t*-tests with Welch’s as described in the figure legends, *p* values were calculated for comparisons between only two groups. Comparisons across more than two groups were made using a two-way ANOVA for more than one independent variable. A Bonferroni post-test was used following significance with an ANOVA. The standard error of the mean is indicated by error bars for each group of data. Differences were considered significant at *P* values below 0.05. All data were analyzed with GraphPad Prism software (San Diego, CA, USA). 

## 3. Results

### 3.1. The mTBI-J Treatment Does Not Induce Severe Cerebral Damage in Juvenile Rats

Animals were subjected to either sham, mTBI-J, or severe-TBI treatment at six weeks old. They were then returned to their home cage and stayed there for 24 h, then sacrificed for TTC stain (all three groups) or brain edema assessment (sham and mTBI-J groups only) (Figure 1). The TTC stain and the brain edema assessment were applied to evaluate the mTBI-J treatment induce cerebral damage in juvenile rats. The severe-TBI served as a corresponding control for comparing the mTBI-J induced brain damage.

**Figure 2:**
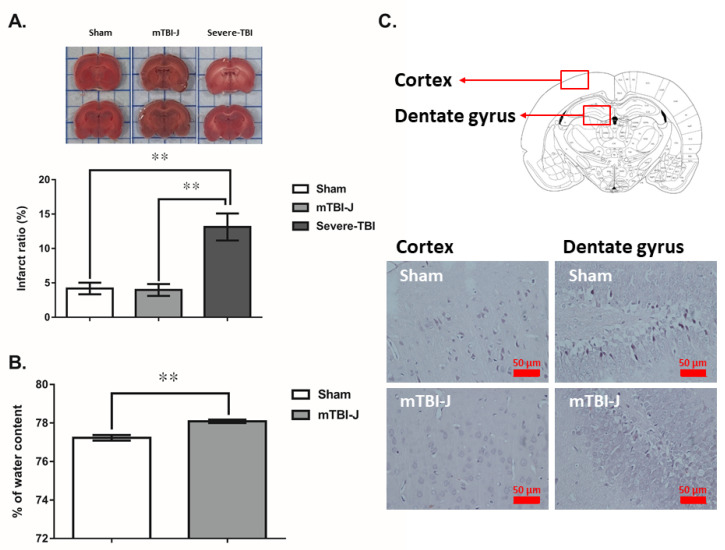
Evaluation of the mTBI-J induced cerebral damage and brain edema. (**A**) Animals are divided into sham, juvenile mild traumatic brain injury treated (mTBI-J), and severe traumatic brain injury. The percent of the total white area was a significant increase in the severe-TBI group, and no significant difference was between mTBI-J and sham control groups. (**B**) The significant difference in the edema between sham and after mild traumatic brain injury 24 h. (**C**) Results of the H&E stain did not reveal significant neural damage in the cerebral cortex and hippocampus. Values are shown as mean ± SEM, ** = *p* < 0.01 compared with the sham control group.

The results of TTC stain showed a significant increase in the area of infarction on severe-TBI treated animals (unpaired *t*-test, two tail, *t* = 4.102, df = 6.915, *p* = 0.0047, *N* = 7 and 6 for sham and severe-TBI group, and unpaired *t*-test, two tail, *t* = 4.268, df = 6.792, *p* < 0.004, *N* = 7 and 6 for mTBI-J and severe-TBI group), but not on mTBI-J and sham groups (unpaired *t*-test, two tail, *t* = 0.256, df = 11.99, *p* = 0.8027, *N* = 7 and 7 for sham and mTBI-J) (Figure 2A). An acute brain edema was found in mTBI-J treated animals comparing with the control group (unpaired *t*-test, two tail, *t* = 5.15, df = 14.78, *p* < 0.001, *N* = 10 for sham and mTBI-J group) (Figure 2B). The H&E stain result did not reveal significant neural damage in the cerebral cortex and hippocampus (Figure 2C).

### 3.2. The Mild Juvenile TBI Treated Animals Showed an Increase in Depression-like Behavior in Adulthood

It is well-documented that adult TBI treatment increases the susceptibility to have depression [28,30,66,67]. Here we examined whether mTBI-J treatment could elevate depression-like behavior in adulthood. Additional sham and mTBI-J treated rats were prepared then subjected to either sucrose preference test (SPT) or forced swimming test (FST) at the age of nine weeks old for evaluating their depression-like behavior (Figure 1). 

**Figure 3:**
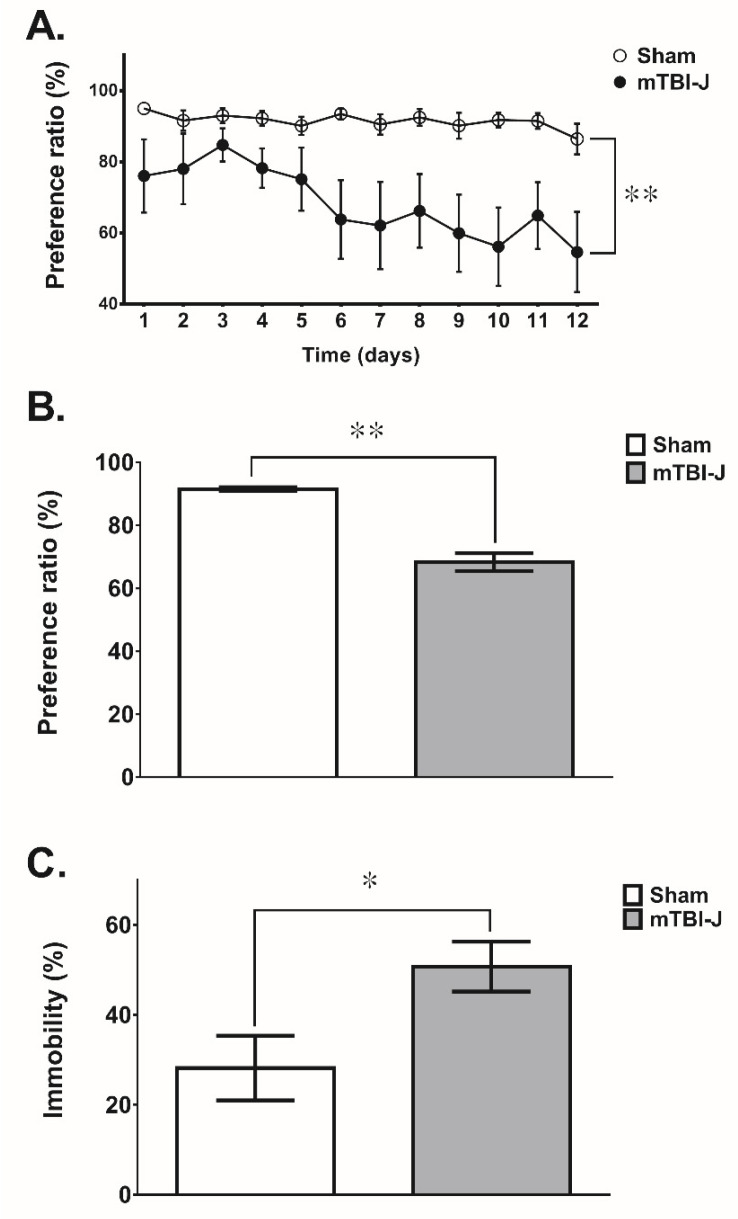
Examine the depression-like behavior in the mTBI-J rats. (**A**) The preference ratio of the mTBI-J group in the sucrose preference test was decreased compared with the sham group (two-way ANOVA, *p* = 0.0079). (**B**) The average preference ratio was summarized, and a significant difference was found among the mTBI-J and sham control groups. (**C**) The immobility percentage in the forced swimming test was significantly increased in the mTBI-J animals. Data was presented as a mean ± SEM (* = *p* < 0.05, ** = *p* < 0.01).

The SPT and the FST results revealed a significant increase in depression-like behaviors in the mTBI-J animals compared with the corresponding sham control groups. In the SPT, the sucrose preference ratio was significantly decreased in the mTBI-J animals compared with the sham control group (two-way ANOVA, F(1, 16) = 9.223, *p* = 0.0079, *N* = 9 per group) (Figure 3A). We further analyzed the average sucrose preference ratio, which decreased by 23% in the mTBI-J animals compared with the sham control group (unpaired *t*-test, two tail, *t* = 8.042, df = 12.06, *p* < 0.0001, *N* = 9 per group) (Figure 3B). In the FST, the percent immobility was increased by 57% in the mTBI-J animals comparing with the sham group (unpaired *t*-test, two tail, *t* = 2.481, df = 14.99, *p* = 0.0254, *N* = 9 per group) (Figure 3C).

### 3.3. Determination of the BDNF and TrkB Expression in the Dorsal Hippocampus and Ventral Hippocampus of mTBI-J Treated Animals

**Figure 4:**
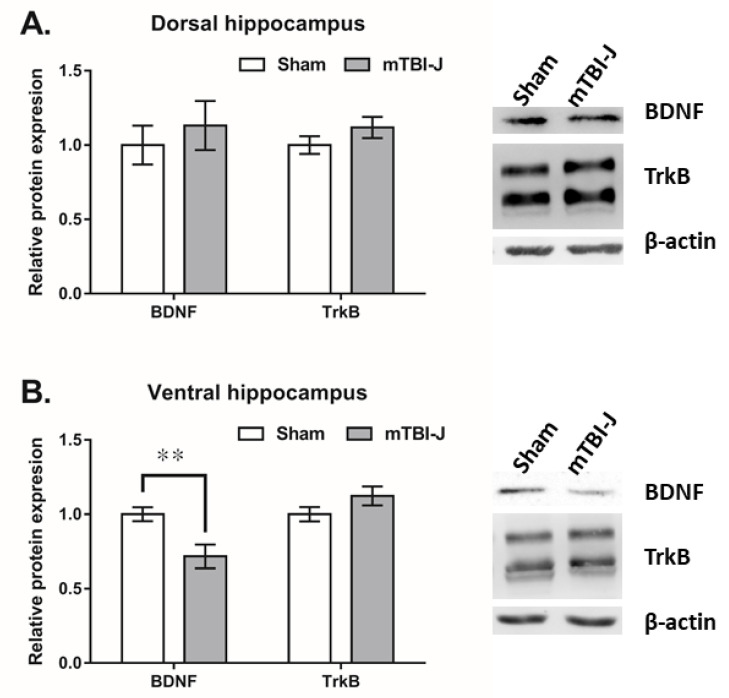
The hippocampal expression of BDNF and TrkB of the mTBI-J treated animals. (**A**) No significant difference was found between the BDNF and TrkB in the dorsal hippocampus of mTBI-J group and sham group (**B**) The expression of BDNF but not TrkB was decreased in the ventral hippocampus of the mTBI-J treated animals. Values are shown as mean ± SEM, ** = *p* < 0.01 compared with the sham control group.

Both BDNF and TrkB have been involved in the depression-like behavior of adult TBI-treated rats or mice [68,69,70]. We performed a western blot to evaluate BDNF and TrkB expression in the ventral hippocampus (vHip) and dorsal hippocampus (dHip). Briefly, rats were sacrificed at nine weeks old following behavioral experiments. Results showed that only the BDNF in vHip was significantly decreased in the mTBI-J treated animals (unpaired *t*-test, two tail, *t* = 3.094, df = 11.39, *p* = 0.01, *N* = 8 per group), but no significant difference was found between the TrkB in the mTBI-J group and sham group (unpaired *t*-test, two tail, *t* = 1.062, df = 13.45, *p* = 0.3071, *N* = 8 per group) (Figure 4B). No significant changes in the BDNF and TrkB were found in the dHip (unpaired *t*-test, two-tailed, *t* = 0.7729 and 0.888, df = 13.55 and 11.04, *p* = 0.4529 and 0.3935, *N* = 8 per group) (Figure 4A).

### 3.4. To Evaluate the Possible Therapeutic Effect of 7,8-DHF on the mTBI-J Induced Depression-like Behavior

For evaluating the possible therapeutic effect of TrkB agonist 7,8-DHF, additional mTBI-J treated rats were prepared and randomly assigned to mTBI-J + vehicle group, or mTBI-J + 7,8-DHF (5 mg/kg i.p.) treated group [54]. The procedure is summarized in Figure 1B. Animals received their first 7,8-DHF injection immediately after the mTBI-J treatment, once a day for a total of twenty-eight days (Figure 1A).

**Figure 5:**
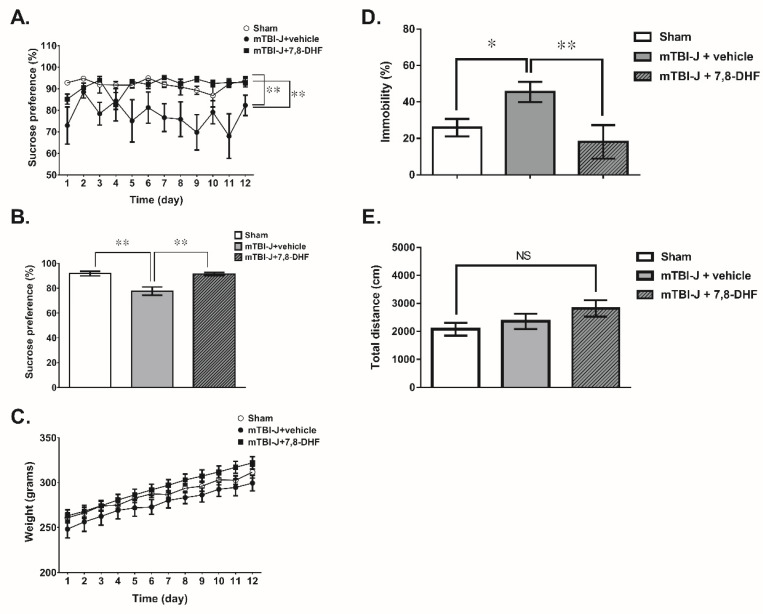
Chronic systemic administration of 7,8-DHF lessens the depression-like behavior of mTBI-treated animals. (**A**) The preference ratio of the mTBI-J+vehicle group in the sucrose preference test was decreased compared with the sham group (two-way ANOVA, *p* = 0.0016), and the 7,8-DHF treatment was restored the sucrose preference ratio compared with the corresponding mTBI-J+vehicle group animals (two-way ANOVA, *p* = 0.0011). (**B**) The average preference ratio was summarized. (**C**) The body weight did not differ between both groups. (**D**) The immobility in the forced swimming test was significantly increased in the mTBI-J+vehicle group animals, but the 7,8-DHF treatment has reduced the immobility. (**E**) There was no difference in the locomotor motor distance in both groups (unpaired *t*-test, *p* = 0.4386, *N* = 10 per sham and mTBI-J+7,8-DHF group groups). Data was showed as mean ± SEM (NS: no significance, * = *p* < 0.05, ** = *p* < 0.01).

Results showed the sucrose preference ratio was restored after 7,8-DHF treatment (two-way ANOVA, F(1, 18) = 14.94, *p* = 0.0011, *N* = 10 per mTBI-J + vehicle group and mTBI-J + 7,8-DHF group (Figure 5A). The average sucrose preference ratio shows the same result, which increased by 13% in the mTBI-J + 7,8-DHF animals compared with the mTBI + vehicle group (unpaired *t*-test, two-tailed, *t* = 3.866, df = 11.42, *p* = 0.0025, *N* = 10 per group) (Figure 4B). There was no difference in body weight between both groups (Figure 4C).

In addition, the percent time of immobility was significantly reduced in the 7,8-DHF treated group compared to the corresponding control animals (two-way ANOVA, F(1, 16) = 9.223, *p* = 0.0079, *N* = 10 per mTBI-J + vehicle group and mTBI-J + 7,8-DHF group (Figure 5D). Furthermore, there was no difference in the locomotor motor distance (unpaired *t*-test, two tail, *t* = 1.151, df = 17.88, *p* = 0.265, *N* = 10 per mTBI + vehicle group and mTBI-J + 7,8-DHF group) (Figure 5E), that was analyzed for excluding the possible non-specific effect of mTBI treatment on the animals’ behavior, which could be misinterpreted as an increase of depression-like behavior. Collectively, the 7-DHF treatment effectively reduced depression-like behavior in the mTBI-J treated animals.

## 4. Discussion

Our results demonstrated that juvenile mild traumatic injury (mTBI-J) treatment did not induce severe neural damage, which could have a long-term adverse neuropsychological impact. Here, an increase the depression-like behaviors in the adulthood of the mTBI-J treated rats was observed. The western blotting data revealed a reduction of BDNF in the ventral hippocampus but not in the dorsal hippocampus. Most importantly, a chronic administration of TrkB agonist 7,8-DHF lessened the depression-like behavior of mTBI-J treated rats at the adult age. In summary, our results suggest the potential usage of 7,8-DHF as a therapeutic agent for preventing the long-term adverse effect of mTBI-J. 

Previous studies have focused mainly on severe TBI inadulthood [37,38,39]. Given the higher incidence of mild TBI (mTBI) at the age of 10~29 [71], it is therefore in the urgent interest of public health that clinical researchers focus on elucidating the pathological mechanisms of, and devising new therapies for, the neurological, psychological consequences of juvenile mild-TBI (mTBI-J). Severe TBI pathophysiology in adult rats was amplified by secondary injuries including hypoxia, ischemia, increased intracranial pressure, seizure, and cerebral edema [22]. The mTBI did not induce significant neural damage; no significant signs of neural damage were found in the TTC staining and H&E staining results. Acute brain edemas were found 24 h after mTBI-J treatment. In comparison to the adult severe TBI treatment that resulted in approximately 40% of mortality in rats, all the mTBI-J treated animals survived without showing sequels, such as motor dysfunction or discoordination. Our previous results showed an increase in hippocampal cation transporter NKCC1 expression, which plays an essential role in the secondary injuries of TBI [21,72]. We did not find a significant increase of NKCC1 in the hippocampus (data not shown). The mechanism underlying and the significance of mTBI-J induced brain edema remains uncertain. Subsequent experiments using glucocorticoids to suppress the brain edema will help elucidate its role on the mTBI-J induced long-term adverse effect. 

Meanwhile, there is evidence that cerebral edema, regardless of the reasons it may be caused (mild trauma, hypoxia/ischemia, moderate neurotoxicity of glutamate), always develops against the background of oxidative and nitrosative stress [73], which leads to the inhibition of the mitochondrial electron transport chain [74]. Energy deficiency leads to the disruption and work of Na + / K + -ATP-ase and Ca2+ -ATP-ase, the disruption of ion homeostasis, and the intracellular increase in the concentration of Na + and Ca2 + ions [75]. An increase in the concentration of Na + ions always leads to an increase in the content of H_2_O molecules, and an increase in the concentration of Ca2+ ions leads to the activation of constitutive NO-synthases (neuronal and endothelial), which in turn leads to the activation of the nitric oxide cycle [76] and, to a greater extent, to a decrease in the energy function of mitochondria [74,75,76]. Thus, with traumatic brain injury of varying severity, negative and positive feedback mechanisms are activated. In the first case, the regulatory processes are not disturbed. In this case, compensatory-adaptive mechanisms are activated that prevent the development of structural damage, and mild traumatic brain injury ultimately leads to the restoration of violations and injuries [77]. In the second case, irreversible violations occur. Against the background of oxidative and nitrosative stress, a typical pathological process develops [78] with its nonspecific pathological reactions characteristic not only of moderate and severe traumatic brain injury [79]. Such processes are universal in nature and develop during hypoxia/ischemia, moderate neurotoxicity of glutamate [73,74,75,77,78], and also participate in the development of the pathogenesis of neurological diseases and mental disorders [80] with long-term consequences as in patients after brain injuries of mild to moderate severity [79,81], and in patients who have been infected with Covid-19 [80,82].

Two categories of behavioral models, learned helplessness and anhedonia, are widely used to study the pathological mechanism of major depressive disorder (MDD) [83,84,85]. We used the FST (for evaluating learned helplessness) and SPT (for examining anhedonia) to measure the depression-like behavior of the mTBI-J animals. Our studies showed the mTBI-J animals’ inability to experience pleasure from rewarding (in SPT) and became more despaired, a core symptom of depression (in FST). Both animal and human studies report impaired sensorimotor function following TBI, and such impairment could be misinterpreted as depression-like behavior. Therefore, the locomotor activity was also measured. Results showed no significant difference in the total moving distance between mTBI-J treated and sham control animals (Figure 5E). Accordingly, the FST showed increased free-floating behavior in the mTBI-J animals is an accurate indicator of depression-like behavior rather than merely reflective of motor deficiency. These results are consistent with past literature on TBI and depression.

Both the dorsal hippocampus (dHip) and ventral hippocampus (vHip) were dissected and subjected to western blot for measuring the expression of BDNF and TrkB. Neither the dHip nor the vHip showed changes in TrkB expression, but only the expression of BDNF was significantly reduced in vHip. The hippocampus is not a homogeneous brain area. It is widely accepted that the dHip and vHip are related to learning and emotion, respectively [86]. A previous study suggested that focal brain damage (post-stroke or posttraumatic) induced hippocampus lesion is essential for the onset of TBI-induced depression-like response [87].

BDNF is one of the most widely distributed and extensively studied neurotrophic in the mammalian brain. Among its functions, one can mention control of neuronal and glial development, neuroprotection, and modulation of both short- and long-lasting synaptic interactions, which are critical for cognition and memory [88]. Tropomyosin receptor kinase B (TrkB) is the receptor of BDNF. It is proven that the BDNF-TrkB pathway plays a critical role in the occurrence of mental illnesses, such as depression and anxiety [89,90,91]. Previous studies suggested that MDD may result from the abnormal expression of hippocampus expression of B induced by acute and chronic stress [91]. The MDD severity inversely correlated to the expression of hippocampal and amygdaloid TrkB. Antidepressants efficacy correlates positively with BDNF expression [49] and the downstream signaling via BDNF and TrkB. These results suggest that BDNF plays a crucial role in the pathophysiology of depression and the therapeutic mechanisms of antidepressants [88]. 

Given that the decrease of BDNF expression was found in the mTBI-J treated animals, we examined whether an administration of TrkB agonist 7,8-DHF could lessen or prevent the mTBI-J treatment-induced depression-like behaviors. Our pilot experiment results showed no therapeutic effect of 7,8-DHF after acute injection or under the subthreshold dose (data not shown). Further experiments such as using another TrkB agonist or directly injecting 7,8-DHF into the vHip will help verify our findings. 

Our results showed mTBI-J treatment transforms into chronic neuropsychiatric conditions, and its underlying mechanisms are largely unknown. In the previous studies, the BDNF promoter activity was decreased in the amygdala after TBI treatment, although it was suggested that decreased amygdaloid BDNF expression is responsible for the elevated anxiety-like response [92]. Recently, the epigenetic modification of chromatin via histone post-translational modifications and DNA methylation has been reported to mediate the adverse effects of environmental perturbations (stress, post-stroke, and posttraumatic) on gene expression in the brain. We speculate a similar mechanism might also be involved here. Further experiments using chromatin immunoprecipitation and bisulfite sequencing will be helpful to examine this possibility. 

In addition to the hippocampus, other brain regions such as the medial prefrontal cortex (mPFC) and nucleus accumbens (NAcc) also play an essential role in both anhedonia and MDD. Besides the BDNF-TrkB pathway, several genes have been associated with depression, and most are related to the hypothalamic-pituitary-adrenal (HPA) axis [93,94], or 5-HT/dopamine reuptake [95,96]. These observations are also consistent with the monoamine hypothesis of depression, suggesting that dysfunctional monoamine neurotransmission is one of the primary causes of MDD. FK506 binding protein 5 (FKBP5), nuclear receptor subfamily 3 group C member 1 (Nr3c1), and corticotropin-releasing hormone (CRH), are genes essential of maintaining normal HPA axis function. We suggest that the possible impact of mTBI-J treatment on the expression of those genes should be examined in the subsequent experiments.

In conclusion, we examined the hypothesis that the BDNF-TrkB signaling pathway is impaired and causes the elevation of depression-like behavior in mTBI-J treated rats. Our results suggested that mTBI-J treatment has long-term regulation effects on hippocampal BDNF expression, leading to increased depression-like behavior in adulthood. We suggest the potential usage of 7,8-DHF as a therapeutic agent for preventing the long-term adverse effect of mTBI-J treatment.

## Data Availability

All data are available upon request.
